# National Patterns in Prescription Opioid Use and Misuse Among Cancer Survivors in the United States

**DOI:** 10.1001/jamanetworkopen.2020.13605

**Published:** 2020-08-17

**Authors:** Vikram Jairam, Daniel X. Yang, Vivek Verma, James B. Yu, Henry S. Park

**Affiliations:** 1 Department of Therapeutic Radiology, Yale University School of Medicine, New Haven, Connecticut; 2 Department of Radiation Oncology, Allegheny General Hospital, Pittsburgh, Pennsylvania; 3 Cancer Outcomes, Public Policy, and Effectiveness Research Center, Yale School of Medicine, New Haven, Connecticut

## Abstract

**Importance:**

Prescription opioids are frequently prescribed to treat cancer-related pain. However, limited information exists regarding rates of prescription opioid use and misuse in populations with cancer.

**Objectives:**

To estimate the prevalence and likelihood of prescription opioid use and misuse in adult cancer survivors compared with respondents without cancer and to identify characteristics associated with prescription opioid use and misuse in adult cancer survivors.

**Design, Setting, and Participants:**

This cross-sectional study is a retrospective, population-based study using data from 169 162 respondents to the National Survey on Drug Use and Health from January 2015 to December 2018. Survey data sets were queried for all respondents aged 18 years or older. Those with a reported history of cancer were termed *cancer survivors* and further divided into *more recent* (had cancer within 12 months of survey) and *less recent* (had cancer more than 12 months prior to survey) cohorts. Respondents with nonmelanoma skin cancer were excluded.

**Main Outcomes and Measures:**

Prescription opioid use and misuse within the past 12 months.

**Results:**

Among 169 162 respondents, 5139 (5.2%) were cancer survivors, with 1243 (1.2%) and 3896 (4.0%) reporting having more recent and less recent cancer histories, respectively. Higher rates of prescription opioid use were observed among more recent cancer survivors (54.3%; 95% CI, 50.2%-58.4%; odds ratio [OR], 1.86; 95% CI, 1.57-2.20; *P* < .001) and less recent cancer survivors (39.2%; 95% CI, 37.3%-41.2%; OR, 1.18; 95% CI, 1.08-1.28; *P* < .001) compared with respondents without cancer (30.5%, reference group). Rates of prescription opioid misuse were similar among more recent (3.5%; 95% CI, 2.4%-5.2%; OR, 1.27; 95% CI, 0.82-1.96; *P* = .36) and less recent (3.0%; 95% CI, 2.4%-3.6%; OR, 1.03; 95% CI, 0.83-1.28; *P* = .76) survivors compared with respondents without cancer (4.3%, reference group). Younger age (aged 18-34 years vs ≥65 years: OR, 7.06; 95% CI, 3.03-16.41; *P* < .001), alcohol use disorder (OR, 3.22; 95% CI, 1.45-7.14; *P* = .005), and nonopioid drug use disorder (OR, 14.76; 95% CI, 7.40-29.44; *P* < .001) were associated with prescription opioid misuse among cancer survivors.

**Conclusions and Relevance:**

In this study, prescription opioid use was higher among more and less recent cancer survivors compared with the population without a history of cancer. Rates of prescription opioid misuse were low and similar among all 3 cohorts. These findings suggest that higher prescription opioid use among cancer survivors may not correspond to increased short-term or long-term misuse.

## Key Points

**Question:**

How do patterns in prescription opioid use and misuse among cancer survivors compare with those among populations without cancer?

**Findings:**

In this cross-sectional study of 169 162 adult respondents, cancer survivors with an incidence of cancer or cancer treatment in the past year had a nearly 2-fold higher likelihood of prescription opioid use compared with respondents without cancer; prescription opioid use decreased among long-term cancer survivors but remained higher compared with the cohort without cancer. Prescription opioid misuse was similar among cancer survivors compared with respondents without cancer.

**Meaning:**

In this study, cancer survivors reported higher rates of prescription opioid use compared with respondents without cancer, but this finding may not translate to increased opioid misuse at a given time.

## Teaser

This cross-sectional study of results from the National Survey on Drug Use and Health compares patterns of opioid use and misuse among cancer survivors with respondents without a history of cancer.

## Introduction

Cancer-related pain is a common complication among cancer survivors and can negatively affect quality of life.^1^ As many as 60% of patients with cancer undergoing active treatment and 33% of cancer survivors may experience cancer-related pain.^1,2,3,4^ Recent reports have also shown higher prescription rates for opioids among cancer survivors compared with the general population.^5,6,7,8,9^ As cancer treatments advance, survival rates will increase accordingly. The American Cancer Society estimates that there were more than 17 million US residents alive with cancer in 2019, with a projected increase to 22 million by 2030.^10^ Therefore, there is a growing need for health care professionals to optimize symptom management for cancer survivors.

Prescription opioids are often used for the treatment of cancer-related pain. While effective at treating moderate to severe cancer-related pain,^3^ opioids also have the potential to be highly addictive. Severe cases of opioid abuse may lead to overdose and death. This fact is underscored by the current nationwide opioid epidemic, in which more than 47 000 people died of opioid-related overdoses in 2017.^11^ Although some studies suggest cancer survivors may have a reduced risk of opioid-related death compared with their counterparts without cancer^12,13^ and that they adhere to their prescribed opioid regimen,^14^ it is unclear how prescription opioid use compares between cancer survivors and patients without cancer. Moreover, reports have shown that a subset of cancer survivors may be at risk for prescription opioid misuse,^15,16,17^ although these studies have generally been conducted in specific populations or in single-institution settings. A national analysis of prescription opioid use and misuse among cancer survivors could serve to inform opioid-prescribing practices by health care professionals in oncology treatment fields.

This study provides a comprehensive analysis of prescription opioid use and misuse among adult cancer survivors. The aims of this study were to identify the prevalence of prescription opioid use and misuse among cancer survivors, identify factors associated with prescription opioid use and misuse, and compare rates of opioid use and misuse between cancer survivors and the population without cancer.

## Methods

### Data Source and Population

The National Survey on Drug Use and Health (NSDUH), conducted by the Substance Abuse and Mental Health Data Archive, is a large national survey on the use of illicit drugs, alcohol, and tobacco as well as on mental health issues among US civilians aged 12 years or older.^18^ Each year, approximately 70 000 individuals are randomly selected across the US to participate, excluding unhoused persons not in shelters, military personnel on active duty, and institutionalized residents (ie, individuals in jails and hospitals). The NSDUH uses a stratified, multistage area probability design that provides estimates at both the state and national level using sample weights.^19^ The NSDUH underwent a partial redesign in 2015 with the implementation of new measures, which included the introduction of specific questions on cancer history and type of cancer that were absent from previous versions of the NSDUH. Therefore, survey data prior to 2015 were excluded from analysis. This study followed Strengthening the Reporting of Observational Studies in Epidemiology (STROBE) reporting guideline for cross-sectional studies.^20^ The Yale Human Investigations Committee granted this study an institutional review board exemption and requirements for informed consent were waived because the data was publicly available and deidentified.

**Figure 1.**
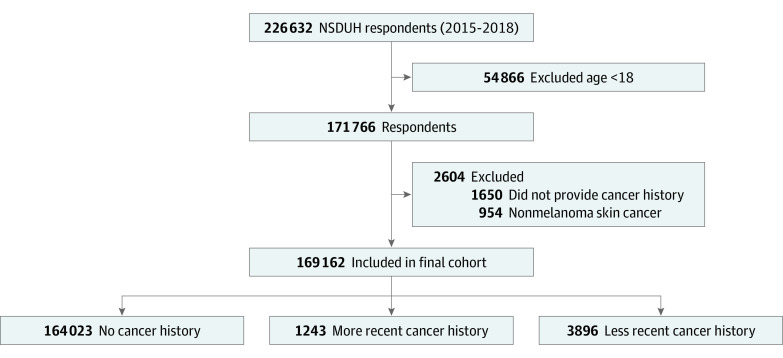
Flow Diagram of Inclusion and Exclusion Criteria for Study

The 2015 to 2018 NSDUH data sets were queried for all respondents aged 18 years or older. Respondents were asked whether they had ever been told they had cancer by a doctor or other health care professional. Participants responding yes were termed cancer survivors and were further divided by recency of cancer history into more recent (had cancer within 12 months of survey) and less recent (had cancer more than 12 months prior to survey) cancer history cohorts. For each respondent, comorbidity history was recorded based on whether the participant reported having certain health conditions. For each cancer survivor, the type of cancer was recorded. Respondents with nonmelanoma skin cancers were excluded because of their low likelihood of requiring opioids for cancer-related pain. A flow diagram depicting the inclusion and exclusion criteria is shown in Figure 1.

*Prescription opioid use* was defined as the use of any prescription opioid in the last 12 months as directed by a doctor. *Prescription opioid misuse* was defined as the use of any prescription opioid in the last 12 months in any way not directed by a doctor. This category included respondents who were prescribed opioids by physicians but did not use them as instructed (eg, using greater doses, higher frequencies, or a longer duration than prescribed) as well as those who were not prescribed opioids but received prescription opioids from other sources (eg, friends or relatives). Prescription opioid misuse also included respondents who, within the past 12 months, met the criteria for prescription opioid abuse or dependence as defined by the American Psychiatric Association *Diagnostic and Statistical Manual of Mental Disorders* (Fourth edition).

### Statistical Analysis

Baseline sociodemographic and clinical characteristics were compared between cancer survivors and respondents without cancer using the Pearson χ^2^ test. Weighted prevalence estimates of prescription opioid use and misuse were stratified by cancer type. Unadjusted and adjusted rates of prescription opioid use and misuse were compared between cancer survivors and participants without cancer using the Pearson χ^2^ test and multivariable logistic regression models, respectively. A second set of multivariable models was created to identify factors associated with prescription opioid use and misuse among cancer survivors. Models were adjusted for age, sex, race, survey year, education level, insurance status, income, urban/rural status, employment status, marital status, self-reported health status, depression history, alcohol use disorder, and nonopioid drug use disorder. Evaluation of depression history, alcohol use disorder, or nonopioid drug use disorder were limited to the 12 months prior to the survey. Adjusted rates of prescription opioid use and misuse over time were also estimated, with estimated margins used to define prevalence. To account for multiple comparisons, a Bonferroni correction was applied, based on the 2 primary outcome variables (prescription opioid use and misuse), with an adjusted significance threshold for 2-sided *P* < .025 (ie, .05 / 2). Sample weighting was used in all models to account for the complex survey design of the NSDUH and to obtain nationally representative estimates. Weighted interview response rates ranged from 66% to 69% between 2015 and 2018. Data analysis was carried out using Stata MP version 16.0 (StataCorp LP).

## Results

### Baseline Characteristics

Among 169 162 respondents, 164 023 (94.8%) had no cancer history and 5139 (5.2%) reported having a cancer history. Participants with a cancer history included 1243 (24.2%) with a more recent cancer history and 3896 (75.8%) with a less recent cancer history. In the entire cohort, 51.8% (90 670) were women, 50.2% (64 746) were between 35 and 64 years old, and 64.0% (102 203) were White patients. The most common cancer types among the 5139 cancer survivors were breast (1118 respondents [24.9%]), prostate or testis (621 respondents [15.6%]), and melanoma (502 respondents [9.5%]). Respondents with a history of cancer had a lower prevalence of prescription opioid use (42.8%; 95% CI, 41.2%-44.5%) compared with those with liver cirrhosis (56.4%; 95% CI, 49.3%-63.1%), chronic obstructive pulmonary disease (52.6%; 95% CI, 50.6%-54.6%), or kidney disease (50.5%; 47.9%-53.1%) (eTable 1 in the Supplement). The prevalence of prescription opioid misuse among cancer survivors was the second lowest (3.1%; 95% CI, 2.7%-3.6%) for all comorbidities except diabetes (2.9%; 95% CI, 2.6%-3.3%).

Baseline characteristics of respondents with and without a cancer history are shown in eTable 2 in the Supplement. Compared with respondents without a history of cancer, respondents with a cancer history were more likely to be older (age ≥65 years: 53.2%; 95% CI, 51.7%-54.9% vs 17.7%; 95% CI, 17.3%-18.1%), to be women (60.2%; 95% CI, 58.2%-62.2% vs 51.3%; 95% CI, 51.0%-51.7%), to be White patients (81.2%; 95% CI, 79.8%-82.5% vs 63.0%; 95% CI, 62.4%-63.5%), to have public health insurance (63.8%; 95% CI, 62.1%-65.5% vs 34.5%; 95% CI, 34.1%-34.9%), and to be married (59.8%; 95% CI, 57.8%-61.8% vs 51.3%; 95% CI, 50.8%-51.8%) (*P* < .001 for all). Participants with a cancer history were less likely to report having alcohol use disorder (3.0%; 95% CI, 2.5%-3.6% vs 6.1%; 95% CI, 6.0%-6.3%) and nonopioid drug use disorder (1.4%; 95% CI, 1.1%-1.8% vs 2.7%; 95% CI, 2.6%-2.8%) compared with respondents without cancer (*P* < .001 for all). A comparison of characteristics between more recent and less recent cancer survivors is shown in eTable 3 in the Supplement.

### Clinical and Sociodemographic Determinants of Prescription Opioid Use and Misuse

Prescription opioid use was higher among respondents with a more recent (54.3%; 95% CI, 50.2%-58.4%) and less recent (39.2%; 95% CI, 37.3%-41.2%) cancer history compared with respondents without cancer (30.5%; 95% CI, 30.2%-30.9%) (*P* < .001) (Table 1). Prescription opioid misuse was lower among respondents with a less recent cancer history compared with participants without cancer (3.0%; 95% CI, 2.4%-3.6% vs 4.3%; 95% CI, 4.2%-4.3%; *P* < .001). On multivariable analysis, cancer survivors with a more recent (OR, 1.86; 95% CI, 1.57-2.20; *P* < .001) and less recent (OR, 1.18; 95% CI, 1.08-1.28; *P* < .001) cancer history had a higher odds of prescription opioid use compared with respondents without a history of cancer. However, neither more recent (OR, 1.27; 95% CI, 0.82-1.96; *P* = .36) nor less recent (OR 1.03; 95% CI, 0.83-1.28; *P* = .76) cancer history was associated with a significant difference in rates of prescription opioid misuse when compared with respondents without a history of cancer.

**Table 1.** Unadjusted and Adjusted Analysis of Prescription Opioid Use or Misuse by Reported Cancer History

Cancer history	Prescription opioid use				Prescription opioid misuse			
	Weighted % (95% CI)	*P* value^a^	OR (95% CI)	*P* value^b^	Weighted % (95% CI)	*P* value^a^	OR (95% CI)	*P* value^b^
No cancer history	30.5 (30.2-30.9)	NA	1 [Reference]	NA	4.3 (4.2-4.4)	NA	1 [Reference]	NA
More recent cancer history	54.3 (50.2-58.4)	<.001	1.86 (1.57-2.20)	<.001	3.5 (2.4-5.2)	.339	1.27 (0.82-1.96)	.36
Less recent cancer history	39.2 (37.3-41.2)	<.001	1.18 (1.08-1.28)	<.001	3.0 (2.4-3.6)	<.001	1.03 (0.83-1.28)	.76

Abbreviation: NA, not applicable; OR, odds ratio.

^a^ *P *values calculated from *χ^2^* comparisons of rates of prescription opioid use and misuse by reported cancer history.

^b^ Multivariable models adjusted for age, sex, race, survey year, education level, insurance status, income, urban/rural status, employment status, marital status, self-reported health status, depression history, alcohol use disorder, and nonopioid drug use disorder.

Among cancer survivors, notable factors associated with prescription opioid use included younger age (ages 35-64 years vs ≥65 years: OR, 1.49; 95% CI, 1.15-1.93; *P* = .003), poorer health status (poor vs excellent: OR, 4.35; 95% CI, 3.00-6.32; *P* < .001), and experiencing a major depressive episode within the past year (OR, 1.52; 95% CI, 1.19-1.95; *P* = .001) (Table 2). Nonopioid drug use disorder was associated with decreased likelihood of prescription opioid use (OR, 0.39; 95% CI, 0.21-0.74; *P* = .005). Factors associated with prescription opioid misuse included younger age (age 18-34 years vs ≥65 years: OR, 7.06; 95% CI, 3.03-16.41; *P* < .001), alcohol use disorder (OR, 3.22; 95% CI, 1.45-7.14; *P* = .005), and nonopioid drug use disorder (OR, 14.76; 95% CI, 7.40-29.44; *P* < .001).

**Table 2.** Multivariable Analysis for Sociodemographic and Clinical Factors Associated With Prescription Opioid Use and Misuse Among Cancer Survivors

Characteristic	Prescription opioid use		Prescription opioid misuse	
	OR (95% CI)	*P* value	OR (95% CI)	*P* value
Age, y				
≥65	1 [Reference]	NA	1 [Reference]	NA
35-64	1.49 (1.15-1.93)	.003	3.83 (1.79-8.19)	.001
18-34	1.40 (0.97-2.01)	.07	7.06 (3.03-16.41)	<.001
Sex				
Women	1 [Reference]	NA	1 [Reference]	NA
Men	0.96 (0.81-1.14)	.65	1.60 (0.88-2.91)	.12
Race				
White	1 [Reference]	NA	1 [Reference]	NA
African American	1.21 (0.90-1.62)	.21	1.31 (0.52-3.30)	.56
Hispanic	0.68 (0.48-0.96)	.03	1.33 (0.66-2.67)	.41
Other^a^	0.88 (0.64-1.21)	.42	1.04 (0.53-2.07)	.90
Year				
2015	1 [Reference]	NA	1 [Reference]	NA
2016	0.95 (0.80-1.14)	.59	0.97 (0.48-1.95)	.92
2017	0.95 (0.75-1.22)	.70	0.79 (0.45-1.38)	.40
2018	0.86 (0.70-1.05)	.14	1.09 (0.62-1.92)	.77
Education level				
<High school	1 [Reference]	NA	1 [Reference]	NA
High school graduate	1.15 (0.85-1.56)	.35	0.52 (0.26-1.06)	.07
Some college or associates degree	1.53 (1.16-2.02)	.003	0.70 (0.33-1.51)	.36
College graduate	1.30 (0.92-1.84)	.14	0.50 (0.24-1.04)	.06
Health insurance				
Other	1 [Reference]	NA	1 [Reference]	NA
Private	1.01 (0.39-2.61)	.99	0.64 (0.06-7.05)	.71
Public	1.35 (0.53-3.46)	.53	0.86 (0.08-9.57)	.90
Unknown	0.70 (0.24-2.04)	.51	0.83 (0.08-8.84)	.88
Income, $				
<20 000	1 [Reference]	NA	1 [Reference]	NA
20 000-49 999	0.80 (0.61-1.03)	.09	0.95 (0.45-1.99)	.90
50 000-74 999	0.86 (0.66-1.11)	.23	1.35 (0.62-2.95)	.45
≥75 000	0.77 (0.57-1.04)	.09	1.56 (0.75-3.25)	.22
Setting				
Urban	1 [Reference]	NA	1 [Reference]	NA
Rural	1.00 (0.82-1.22)	.99	1.11 (0.66-1.85)	.70
Employment				
Full time	1 [Reference]	NA	1 [Reference]	NA
Part time	1.05 (0.78-1.42)	.75	0.45 (0.23-0.91)	.03
Unemployed	0.73 (0.43-1.26)	.26	1.03 (0.32-3.25)	.96
Other	0.98 (0.77-1.25)	.88	0.98 (0.51-1.89)	.95
Marital status				
Never married	1 [Reference]	NA	1 [Reference]	NA
Married	1.03 (0.78-1.36)	.83	0.86 (0.47-1.58)	.63
Widowed	0.93 (0.69-1.25)	.62	0.78 (0.17-3.56)	.74
Divorced or separated	1.16 (0.85-1.58)	.34	0.88 (0.48-1.63)	.69
Health status				
Excellent	1 [Reference]	NA	1 [Reference]	NA
Very good	1.40 (1.07-1.84)	.02	0.77 (0.32-1.87)	.56
Good	1.90 (1.45-2.49)	<.001	1.04 (0.41-2.65)	.93
Fair	2.42 (1.78-3.29)	<.001	1.23 (0.47-3.26)	.67
Poor	4.35 (3.00-6.32)	<.001	1.36 (0.45-4.10)	.58
Major depressive episode within year	1.52 (1.19-1.95)	.001	2.05 (0.89-4.72)	.09
Missing data	0.68 (0.32-1.45)	.31	0.82 (0.26-2.60)	.73
Alcohol use disorder	0.76 (0.49-1.17)	.21	3.22 (1.45-7.14)	.005
Nonopioid drug use disorder	0.39 (0.21-0.74)	.005	14.76 (7.40-29.44)	<.001

Abbreviation: NA, not applicable; OR, odds ratio.

^a^ Includes Native American and Alaskan Native, Native Hawaiian and Pacific Islander, Asian, and non-Hispanic mixed race.

### Prescription Opioid Use and Misuse by Cancer Type

**Figure 2.**
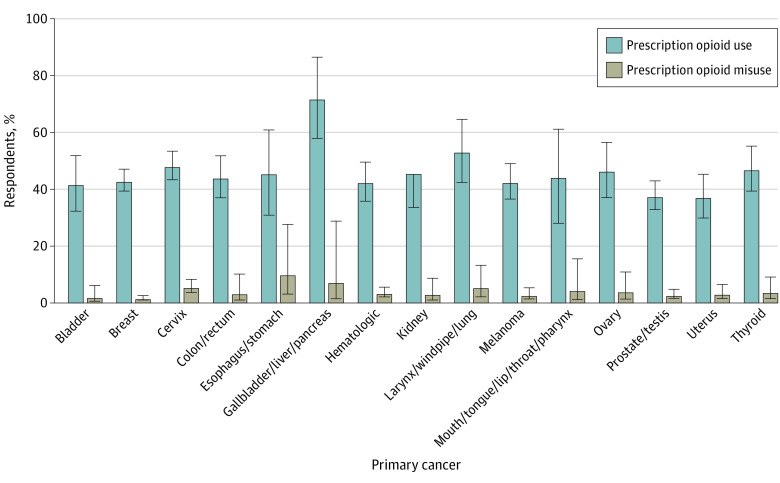
Weighted Prevalence Estimates of Prescription Opioid Use and Misuse by Cancer Type Error bars indicate 95% CIs.

Respondents with gallbladder, liver, or pancreatic cancer (72.2% [44 of 73 respondents]), larynx, windpipe, or lung cancer (53.5% [80 of 160 respondents]), and cervical cancer (48.4% [248 of 509 respondents]) reported the highest rates of prescription opioid use. Those with prostate or testis cancer (37.9% [231 of 621 respondents]) and uterine cancer (37.6% [103 of 240 respondents]) had the lowest rates of opioid use. Meanwhile, prescription opioid misuse was highest among respondents with esophagus or stomach cancer (10.1% [4 of 64 respondents]) and gallbladder, liver, or pancreatic cancer (7.3% [3 of 73 respondents]) and lowest among those with breast cancer (1.7% [26 of 1118 respondents]) (Figure 2).

### Temporal Trends in Prescription Opioid Use and Misuse

**Figure 3.**
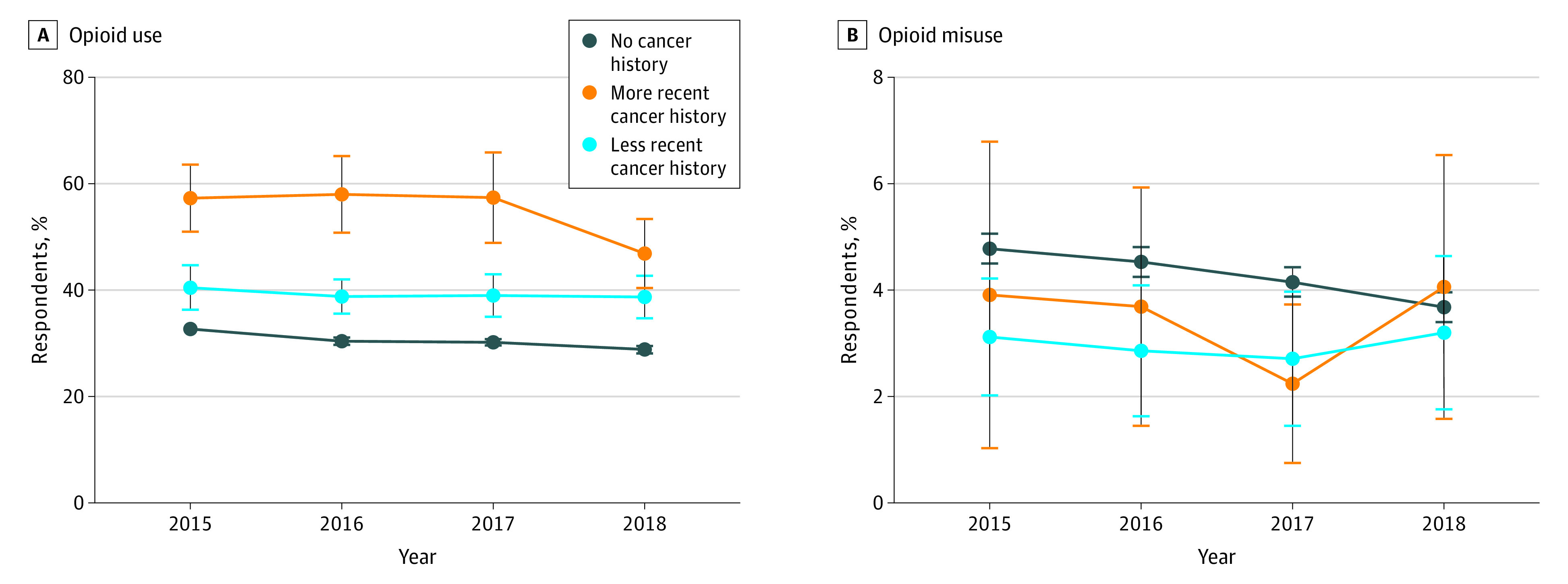
Temporal Trends in Adjusted Prevalence of Prescription Opioid Use and Misuse by Reported Cancer History Error bars indicate 95% CIs.

Prescription opioid use declined 3.9%, from 32.7% (95% CI, 32.0%-33.4%) in 2015 to 28.8% (95% CI, 28.2%-29.5%) in 2018 (*P* < .001) among respondents without cancer (Figure 3). A trend toward decreasing opioid use from 57.3% (95% CI, 50.1%-63.8%) in 2015 to 46.9% (95% CI, 40.3%-53.8%) in 2018 (*P* = .03), representing a decline of 10.4%, was also observed among more recent cancer survivors. The prevalence of prescription opioid misuse declined by 1.1%, from 4.8% (95% CI, 4.5%-5.1%) in 2015 to 3.7% (95% CI, 3.5%-4.0%) in 2018 (*P* < .001) among respondents without cancer and was unchanged among more recent (2015: 3.5%; 95% CI, 1.8%-6.7% vs 2018: 4.3%; 95% CI, 2.5%-7.3%; *P* = .98) and less recent (2015: 3.1%, 95% CI, 2.1%-4.1% vs 2018: 3.2%, 95% CI, 2.0%-5.2%; *P* = .96) cancer survivors.

## Discussion

In this novel examination of national patterns in prescription opioid behavior among cancer survivors, multiple findings emerged. First, prescription opioid use was higher among cancer survivors than among respondents without cancer, with more recent cancer survivors reporting a nearly 2-fold higher rate of use. Second, adjusted rates of prescription opioid misuse were no different among cancer survivors compared with those without a history of cancer. Third, notable factors associated with prescription opioid use or misuse included younger age, cancer type, having a major depressive episode, alcohol use disorder, and nonopioid drug use disorder. Fourth, rates of prescription opioid use and misuse varied by cancer type. Fifth, reported rates of prescription opioid use decreased over time among respondents without cancer but decreased more steeply among more recent cancer survivors.

Patterns in opioid use and misuse among cancer survivors compared with the population without cancer warrant further discussion. We found a nearly 2-fold increase in opioid use among patients with a more recent cancer history, who are likely to be more symptomatic from their disease or treatment-related side effects.^7^ In respondents with less recent cancer histories, opioid use decreased but remained nearly 20% higher compared with the population without cancer. Similar findings were observed by Salz et al,^6^ who found the highest rates of opioid use occurred in the first year after diagnosis of lung and colorectal cancer. However, by 6 years after diagnosis, opioid use decreased to similar levels as control participants without cancer. Our study also showed that adjusted rates of prescription opioid misuse were no different among cancer survivors than among patients without a history of cancer. This suggests that increased prescription opioid use among cancer survivors does not necessarily translate to a higher risk of misuse. Our findings dovetail with a 2020 report^21^ showing a 10-fold lower incidence of opioid-related deaths in cancer survivors compared with the general population. Moreover, we found that the prevalence of opioid misuse among cancer survivors in any given year was relatively low (ranging from 2.2% to 4.1%), consistent with the estimate of 2.9% reported in another study.^17^ Given recent concerns about inadequate opioid prescribing in cancer survivors,^22,23^ our analysis could reassure both patients and professionals who have concerns about opioid misuse or addiction.

Our study found multiple sociodemographic and clinical factors associated with prescription opioid use and misuse among cancer survivors. Younger respondents had higher rates of opioid use and misuse, consistent with multiple studies in populations with and without cancer.^16,24,25^ This highlights the importance of accurately screening for and identifying aberrant opioid use in younger patients, who may experience long-term survivorship. Participants reporting a major depressive episode within the past year had a higher likelihood of opioid use. These patients may be more susceptible to experiencing cancer-related pain, which can be modulated by psychosocial factors.^26^ However, on adjusted analysis, respondents with major depressive episodes were not more likely to misuse opioids. This may be because of the overlap between cancer survivors with depression and those with alcohol or substance use disorders, with the latter 2 being stronger risk factors. Notably, nonopioid drug use disorder was associated with a decreased likelihood of prescription opioid use. This may be because patients are usually screened for substance use disorders prior to treatment with prescription opioids. Oncologists may be less likely to prescribe opioids to patients who screen positive. Finally, comorbid alcohol or nonopioid drug use disorder were among the strongest factors associated with opioid misuse. Our findings underscore the importance of performing a thorough social history to identify underlying mental health or substance use disorders in patients suspected of opioid misuse.

We observed a high rate of prescription opioid use and misuse among respondents with upper gastrointestinal (GI) cancers, including gallbladder, liver, or pancreatic cancer as well as esophagus or stomach cancer. Previous studies^5,17^ have demonstrated elevated rates of opioid prescribing and abuse in survivors of GI cancer using registry data, although this is the first to use self-reported survey data, to our knowledge. There may be multiple reasons for this finding. First, more than half of patients with GI cancers report having chronic pain.^2^ Second, comorbid alcohol or tobacco use disorders are risk factors for developing GI cancers and for long-term opioid use.^27,28^ Our findings also showed that respondents with cervical cancer had high rates of prescription opioid use and misuse. Patients with cervical cancer may be at higher risk of opioid misuse compared with those with other gynecologic malignant neoplasms, possibly because of a higher incidence of chronic pain.^29,30,31^ Similarly, patients with larynx or lung cancer may be at risk for long-term opioid use because of the incidence of advanced disease at diagnosis and need for trimodality therapy.^32^ Meanwhile, low rates of prescription opioid use and misuse were observed among respondents with prostate or testis, uterine, and breast cancer. This may be because of a combination of having more indolent histologies, more effective screening methods in prostate and breast cancer, and a high likelihood of requiring only single or bimodality therapy, thereby reducing long-term toxicity.

The time frame of this study coincided with the passage of legislation and guidelines intended to curb excess opioid prescribing among health care professionals. Such measures included mandatory-access prescription drug monitoring programs and opioid prescription guidelines for chronic pain issued by the US Centers for Disease Control and Prevention in March 2016.^33^ Indeed, our findings showed a numerically larger decline in prescription opioid use (−10.4%) among more recent cancer survivors who are likely to be receiving active treatment, compared with respondents without a history of cancer (−3.9%). This suggests that legislation and guidelines designed to target patients without cancer or protect cancer survivors may be inadvertently affecting cancer survivors. This is consistent with recent data showing that the largest decline in opioid prescriptions among oncology patients occurred in states with cancer-exempt monitoring programs.^34^ Meanwhile, rates of prescription opioid misuse declined by 1.1% among respondents without cancer but did not change appreciably among cancer survivors. The decrease in prescription opioid use by 10% among more recent cancer survivors in the absence of a decrease in misuse may indicate reduced opioid access for cancer survivors who stand to benefit, while ineffectively addressing the issue of opioid misuse. Despite some monitoring programs and the Centers of Disease Control and Prevention guidelines exempting patients with cancer, there is concern that legislation and prescribing recommendations are being inappropriately applied to patients with cancer.^23,35^ One survey study conducted in 2018^36^ of cancer survivors found that 35% of respondents reported that their physician refused to give them an opioid prescription, and nearly half reported their physician told them their pain treatment options were limited by laws, guidelines, or insurance coverage. While caution against opioid misuse is certainly warranted, it should not come at the expense of effective pain management and improved quality of life for cancer survivors.

### Limitations

There are multiple limitations to this study. First, respondents may have limited ability or be unwilling to accurately self-report opioid misuse, resulting in the risk of being underreported in the survey. However, the consistency in the reported rate of misuse between our study and others in the literature imparts a degree of confidence to our findings. Second, the survey lacks detailed information on cancer stage and treatment history, both of which have known associations with long-term opioid use. Third, opioid misuse as defined by the NSDUH comprises a heterogeneous category, so determining clinically meaningful misuse relies on input from the providing physician. Fourth, the NSDUH only allows for temporal dichotomization of cancer history either within a year or more than 1 year since survey administration. This in turn may group cancer survivors who are without evidence of disease for 1 year with those who have been in remission for many years in the less recent cancer history cohort. Nevertheless, to our knowledge, the NSDUH is the only existing data set with the capability to provide a nationally representative analysis of self-reported opioid use and misuse among patients with a cancer history.

## Conclusions

In this study, cancer survivors reported higher rates of prescription opioid use and similar rates of prescription opioid misuse compared with respondents without cancer. These findings suggest that increased prescription opioid use among cancer survivors does not necessarily translate to a higher risk of misuse. It is imperative that opioid legislation and policies recognize that cancer survivors will have a higher rate of prescription opioid use and that restrictions on prescription opioid access for cancer survivors are incongruent with their opioid utilization patterns. Our analysis supports continued access to opioid medications for cancer survivors who may benefit from such therapy.

## Notes

Supplement. **eTable 1. **Weighted Prevalence of Prescription Opioid Use and Misuse by Comorbidity History **eTable 2.** Baseline Characteristics of Adult Population Stratified by Reported Cancer History **eTable 3. **Baseline Characteristics of Adult Cancer Population Stratified by Recency of Cancer History
