# Supplementary material for: National Patterns in Prescription Opioid Use and Misuse Among Cancer Survivors in the United States
Source: JAMA Netw Open. 2020 Aug 17;3(8):e2013605. doi: 10.1001/jamanetworkopen.2020.13605 (PMC7431994; doi:10.1001/jamanetworkopen.2020.13605)
Supplement: Supplement. — eTable 1. Weighted Prevalence of Prescription Opioid Use and Misuse by Comorbidity History eTable 2. Baseline Characteristics of Adult Population Stratified by Reported Cancer History eTable 3. Baseline Characteristics of Adult Cancer Population Stratified by Recency of Cancer History [file jamanetwopen-3-e2013605-s001.pdf]

# Supplementary Online Content

Jairam V, Yang DX, Verma V, Yu JB, Park HS. National patterns in prescription opioid use and misuse among cancer survivors in the United States. *JAMA Netw Open*. 2020;3(8):e2013605. doi:10.1001/jamanetworkopen.2020.13605

**eTable 1.** Weighted Prevalence of Prescription Opioid Use and Misuse by Comorbidity History

**eTable 2.** Baseline Characteristics of Adult Population Stratified by Reported Cancer History

**eTable 3.** Baseline Characteristics of Adult Cancer Population Stratified by Recency of Cancer History

This supplementary material has been provided by the authors to give readers additional information about their work.

© 2020 Jairam V et al. *JAMA Network Open.*

**eTable 1.** Weighted Prevalence of Prescription Opioid Use and Misuse by Comorbidity History

| | Weighted % (95% CI) | |
|---|---|---|
| **Comorbidity** | **Prescription Opioid Use** | **Prescription Opioid Misuse** |
| Asthma | 39.7 (38.5 – 40.9) | 5.2 (4.8 – 5.7) |
| Cancer | 42.8 (41.2 – 44.5) | 3.1 (2.7 – 3.6) |
| Chronic Obstructive Pulmonary Disease | 52.6 (50.6 – 54.6) | 4.8 (4.1 – 5.5) |
| Diabetes | 42.8 (41.7 – 44.0) | 2.9 (2.6 – 3.3) |
| Hepatitis B or C | 42.7 (39.0 – 46.6) | 8.3 (6.8 – 10.1) |
| Heart Condition | 42.1 (40.7 – 43.6) | 3.4 (3.0 – 3.8) |
| HIV or AIDS | 38.5 (31.6 – 45.8) | 10.6 (7.0 – 15.8) |
| Hypertension | 41.2 (40.2 – 42.2) | 3.3 (3.1 – 3.5) |
| Kidney Disease | 50.5 (47.9 – 53.1) | 3.4 (2.7 – 4.4) |
| Liver Cirrhosis | 56.4 (49.3 – 63.1) | 4.9 (2.9 – 8.1) |

Abbreviations: CI, confidence interval

© 2020 Jairam V et al. *JAMA Network Open.*

**eTable 2.** Baseline Characteristics of Adult Population Stratified by Reported Cancer History

| | Weighted % (95% CI) | | |
|---|---|---|---|
| **Characteristics** | **No Cancer History[a] (N = 164,023)** | **Cancer History[a] (N = 5,139)** | **P-Value** |
| **Age** | | | <0.001 |
| 18 to 34 | 31.6 (31.1 – 32.1) | 4.9 (4.6 – 5.4) | |
| 35 to 64 | 50.7 (50.3 – 51.1) | 41.8 (40.2 – 43.3) | |
| 65+ | 17.7 (17.3 – 18.1) | 53.2 (51.7 – 54.9) | |
| **Sex** | | | <0.001 |
| Male | 48.7 (48.3 – 49.0) | 39.8 (37.8 – 41.8) | |
| Female | 51.3 (51.0 – 51.7) | 60.2 (58.2 – 62.2) | |
| **Race** | | | <0.001 |
| White | 63.0 (62.4 – 63.5) | 81.2 (79.8 – 82.5) | |
| African-American | 12.2 (11.8 – 12.6) | 7.1 (6.2 – 8.0) | |
| Hispanic | 16.5 (16.0 – 16.9) | 7.2 (6.4 – 8.1) | |
| Other | 8.3 (8.1 – 8.6) | 4.5 (3.7 – 5.5) | |
| **Year** | | | 0.346 |
| 2015 | 24.7 (24.3 – 25.1) | 24.4 (22.7 – 26.2) | |
| 2016 | 24.8 (24.5 – 25.1) | 24.9 (23.3 – 26.6) | |
| 2017 | 25.1 (24.9 – 25.4) | 24.1 (22.5 – 25.7) | |
| 2018 | 25.2 (24.8 – 25.6) | 26.6 (24.8 – 28.5) | |
| **Education Level** | | | <0.001 |
| Less than high school | 13.0 (12.8 – 13.3) | 9.2 (8.0 – 10.6) | |
| High school graduate | 25.1 (24.7 – 25.4) | 23.8 (22.1 – 25.5) | |
| Some college/Associates Degree | 31.0 (30.6 – 31.4) | 31.5 (29.6 – 33.5) | |
| College graduate | 30.9 (30.3 – 31.4) | 35.5 (33.5 – 37.6) | |
| **Health Insurance** | | | <0.001 |
| Private | 52.9 (52.5 – 53.3) | 32.9 (31.2 – 34.6) | |
| Public | 34.5 (34.1 – 34.9) | 63.8 (62.1 – 65.5) | |
| Other | 2.3 (2.2 – 2.4) | 0.7 (0.4 – 1.0) | |
| Unknown | 10.3 (10.0 – 10.5) | 2.6 (2.2 – 3.1) | |
| **Income** | | | <0.001 |
| <$20,000 | 16.8 (16.4 – 17.1) | 13.5 (12.2 – 14.8) | |
| $20,000 -$49,999 | 29.6 (29.2 – 30.0) | 31.2 (29.6 – 32.9) | |
| $50,000 - $74,999 | 15.9 (15.6 – 16.2) | 17.0 (15.9 – 18.2) | |
| $75,000+ | 37.6 (37.0 – 38.2) | 38.2 (36.4 – 40.1) | |
| **Setting** | | | <0.001 |
| Urban | 85.8 (85.4 – 86.3) | 83.5 (82.3 – 84.6) | |
| Rural | 14.1 (13.7 – 14.5) | 16.5 (15.4 – 17.7) | |
| **Employment** | | | <0.001 |
| Full Time | 50.8 (50.4 – 51.2) | 29.4 (27.5 – 31.3) | |
| Part Time | 13.2 (12.9 – 13.4) | 11.7 (10.7 – 12.8) | |
| Unemployed | 4.6 (4.4 – 4.7) | 1.7 (1.4 – 2.1) | |
| Other | 31.4 (30.9 – 31.8) | 57.1 (55.4 – 58.8) | |
| **Marital Status** | | | <0.001 |

© 2020 Jairam V et al. *JAMA Network Open.*

| | | | |
|---|---|---|---|
| Married | 51.3 (50.8 – 51.8) | 59.8 (57.8 – 61.8) | |
| Widowed | 5.3 (5.1 – 5.6) | 13.6 (12.3 – 15.0) | |
| Divorced/Separated | 13.7 (13.4 – 14.0) | 16.9 (15.5 – 18.5) | |
| Never Married | 29.6 (29.1 – 30.0) | 9.7 (8.8 – 10.6) | |
| **Health Status** | | | <0.001 |
| Excellent | 21.7 (21.3 – 22.1) | 12.6 (11.5 – 13.9) | |
| Very good | 36.2 (35.8 – 36.5) | 29.4 (27.6 – 31.1) | |
| Good | 28.8 (28.3 – 29.2) | 34.2 (32.3 – 36.1) | |
| Fair | 10.8 (10.5 – 11.1) | 16.3 (15.0 – 17.8) | |
| Poor | 2.4 (2.3 – 2.5) | 7.4 (6.4 – 8.6) | |
| Unknown | 0.01 (0.01 – 0.03) | 0 | |
| **Major Depressive Episode within Year** | 6.9 (6.7 – 7.1) | 6.9 (6.1 – 7.8) | 0.966 |
| Unknown | 0.7 (0.7 – 0.8) | 0.7 (0.4 – 1.1) | |
| **Alcohol Use Disorder** | 6.1 (6.0 – 6.3) | 3.0 (2.5 – 3.6) | <0.001 |
| **Non-Opioid Drug Use Disorder** | 2.7 (2.6 – 2.8) | 1.4 (1.1 – 1.8) | <0.001 |
| **Cancer Type**[b] | | | N/A |
| Bladder | - | 3.5 (2.8 – 4.2) | |
| Breast | - | 2.5 (2.3 – 2.6) | |
| Cervix | - | 6.3 (5.6 – 7.1) | |
| Colon/Rectum | - | 7.0 (6.1 – 7.9) | |
| Esophagus/Stomach | - | 1.4 (1.0 – 1.9) | |
| Gallbladder/Liver/Pancreas | - | 1.5 (1.1 – 2.0) | |
| Hematologic | - | 8.3 (7.3 – 9.5) | |
| Kidney | - | 2.7 (2.1 – 3.4) | |
| Larynx/Windpipe/Lung | - | 3.8 (3.0 – 4.7) | |
| Melanoma | - | 9.5 (8.5 – 10.5) | |
| Mouth/Tongue/Lip/Throat/Pharynx | - | 1.5 (1.1 – 1.9) | |
| Ovary | - | 2.9 (2.3 – 3.6) | |
| Prostate/Testis | - | 15.6 (14.2 – 17.1) | |
| Uterus | - | 4.4 (3.8 – 5.2) | |
| Thyroid | - | 4.0 (3.5 – 4.7) | |

Abbreviations: CI, confidence interval

[a]Percentages may not add up to 100 due to rounding.

[b]Percentages add up to > 100 due to respondents reporting multiple cancers

© 2020 Jairam V et al. *JAMA Network Open.*

**eTable 3.** Baseline Characteristics of Adult Cancer Population Stratified by Recency of Cancer History

| | Weighted % (95% CI) | | |
|---|---|---|---|
| **Characteristics** | **More Recent Cancer History[a] (N = 1,243)** | **Less Recent Cancer History[a] (N = 3,896)** | **P-Value** |
| **Age** | | | 0.763 |
| 18 to 34 | 4.6 (3.8 – 5.6) | 5.1 (4.5 – 5.6) | |
| 35 to 64 | 41.6 (38.1 – 45.1) | 41.8 (40.2 – 43.4) | |
| 65+ | 53.8 (50.2 – 57.4) | 53.1 (51.5 – 54.7) | |
| **Sex** | | | <0.001 |
| Male | 49.0 (44.7 – 53.3) | 36.9 (34.7 – 65.2) | |
| Female | 51.0 (46.7 – 55.3) | 63.1 (60.9 – 65.2) | |
| **Race** | | | 0.042 |
| White | 78.8 (75.8 – 81.5) | 81.9 (80.3 – 83.4) | |
| African-American | 9.4 (7.5 – 11.8) | 6.3 (5.4 – 7.4) | |
| Hispanic | 7.4 (5.7 – 9.6) | 7.2 (6.3 – 8.2) | |
| Other | 4.4 (3.1 – 6.2) | 4.5 (3.7 – 5.6) | |
| **Year** | | | 0.012 |
| 2015 | 24.1 (21.1 – 27.4) | 24.5 (22.6 – 26.5) | |
| 2016 | 22.0 (19.2 – 25.1) | 25.8 (23.9 – 27.6) | |
| 2017 | 23.2 (20.4 – 26.3) | 24.4 (22.6 – 26.2) | |
| 2018 | 30.6 (27.3 – 34.1) | 25.3 (23.5 – 27.2) | |
| **Education Level** | | | 0.009 |
| Less than high school | 12.5 (10.0 – 15.4) | 8.2 (7.0 – 9.6) | |
| High school graduate | 24.2 (20.9 – 27.8) | 23.6 (21.8 – 25.5) | |
| Some college/Associates Degree | 29.9 (26.3 – 33.8) | 32.0 (30.1 – 34.0) | |
| College graduate | 33.3 (29.5 – 27.4) | 36.2 (33.8 – 38.5) | |
| **Health Insurance** | | | 0.002 |
| Private | 30.4 (27.2 – 33.7) | 33.7 (31.9 – 35.4) | |
| Public | 67.6 (64.3 – 70.6) | 62.6 (60.9 – 64.3) | |
| Other | 0.8 (0.4 – 1.9) | 0.6 (0.4 – 1.0) | |
| Unknown | 1.1 (0.7 – 2.0) | 3.0 (2.5 – 3.7) | |
| **Income** | | | 0.017 |
| <$20,000 | 16.7 (14.4 – 19.2) | 12.4 (11.0 – 14.1) | |
| $20,000 -$49,999 | 31.8 (28.4 – 35.3) | 31.0 (29.1 – 33.1) | |
| $50,000 - $74,999 | 15.7 (13.2 – 18.6) | 17.4 (16.1 – 18.8) | |
| $75,000+ | 35.7 (32.4 – 39.2) | 39.0 (37.1 – 41.0) | |
| **Setting** | | | 0.857 |
| Urban | 83.6 (80.8 – 86.2) | 83.4 (82.1 – 84.6) | |
| Rural | 16.3 (13.8 – 19.2) | 16.6 (15.4 – 17.9) | |
| **Employment** | | | 0.024 |
| Full Time | 26.2 (22.7 – 30.1) | 30.4 (28.5 – 32.3) | |
| Part Time | 10.4 (8.1 – 13.3) | 12.2 (11.1 – 13.3) | |
| Unemployed | 1.4 (0.8 – 2.4) | 1.8 (1.5 – 2.3) | |
| Other | 61.9 (58.1 – 65.6) | 55.6 (54.0 – 57.2) | |

© 2020 Jairam V et al. *JAMA Network Open.*

| | | | |
|---|---|---|---|
| **Marital Status** | | | 0.399 |
| Married | 57.8 (54.4 – 61.3) | 60.4 (58.1 – 62.6) | |
| Widowed | 14.8 (12.4 – 17.6) | 13.2 (11.7 – 15.0) | |
| Divorced/Separated | 16.8 (14.4 – 19.6) | 16.9 (15.3 – 18.6) | |
| Never Married | 10.4 (9.2 – 11.8) | 9.4 (8.5 – 10.4) | |
| **Health Status** | | | <0.001 |
| Excellent | 5.9 (4.2 – 8.1) | 14.8 (13.4 – 16.2) | |
| Very good | 22.6 (19.2 – 26.3) | 31.5 (29.4 – 33.7) | |
| Good | 32.4 (29.2 – 35.8) | 34.8 (32.5 – 37.1) | |
| Fair | 23.1 (20.9 – 25.4) | 14.2 (12.6 – 16.0) | |
| Poor | 16.0 (12.8 – 19.6) | 4.7 (3.9 – 5.7) | |
| Unknown | 0.01 (0.0 – 0.1) | 0 | |
| **Major Depressive Episode within Year** | 7.6 (5.9 – 9.6) | 6.7 (5.6 – 7.9) | 0.613 |
| Unknown | 0.9 (0.4 – 1.9) | 0.7 (0.4 – 1.2) | |
| **Alcohol Use Disorder** | 3.0 (2.1 – 4.4) | 3.0 (2.4 – 3.8) | 0.977 |
| **Non-Opioid Drug Use Disorder** | 1.1 (0.6 – 1.8) | 1.5 (1.1 – 2.0) | 0.180 |
| **Cancer Type**[b] | | | <0.001 |
| Bladder | 4.8 (3.5 – 6.5) | 3.0 (2.4 – 3.8) | |
| Breast | 17.9 (14.7 – 21.6) | 27.0 (25.1 – 29.1) | |
| Cervix | 3.6 (2.6 – 5.0) | 7.2 (6.3 – 8.1) | |
| Colon/Rectum | 7.3 (5.7 – 9.4) | 6.9 (5.9 – 8.0) | |
| Esophagus/Stomach | 2.0 (1.1 – 3.4) | 1.2 (0.8 – 1.8) | |
| Gallbladder/Liver/Pancreas | 4.0 (2.7 – 5.8) | 0.7 (0.4 – 1.1) | |
| Hematologic | 13.9 (11.2 – 17.1) | 6.6 (5.6 – 7.7) | |
| Kidney | 2.9 (1.8 – 4.8) | 2.6 (2.0 – 3.4) | |
| Larynx/Windpipe/Lung | 6.9 (4.9 – 9.4) | 2.8 (2.2 – 3.6) | |
| Melanoma | 10.4 (8.3 – 13.0) | 9.2 (8.1 – 10.4) | |
| Mouth/Tongue/Lip/Throat/Pharynx | 1.3 (0.7 – 2.1) | 1.5 (1.1 – 2.1) | |
| Ovary | 2.0 (1.1 – 3.4) | 3.2 (2.5 – 3.9) | |
| Prostate/Testis | 18.8 (16.0 – 21.9) | 14.6 (13.1 – 16.3) | |
| Uterus | 2.4 (1.5 – 3.9) | 5.1 (4.4 – 5.9) | |
| Thyroid | 2.2 (1.5 – 3.2) | 4.6 (3.9 – 5.4) | |

Abbreviations: CI, confidence interval

[a]Percentages may not add up to 100 due to rounding.

[b]Percentages add up to > 100 due to respondents reporting multiple cancers

© 2020 Jairam V et al. *JAMA Network Open.*
